# Supplementary material for: Rescue of Escherichia coli auxotrophy by de novo small proteins
Source: eLife. 2023 Mar 15;12:e78299. doi: 10.7554/eLife.78299 (PMC10065794; doi:10.7554/eLife.78299)
Supplement: Figure 3—source data 1. [file elife-78299-fig3-data1.zip › Figure 3B - labeled source data 3.pdf]

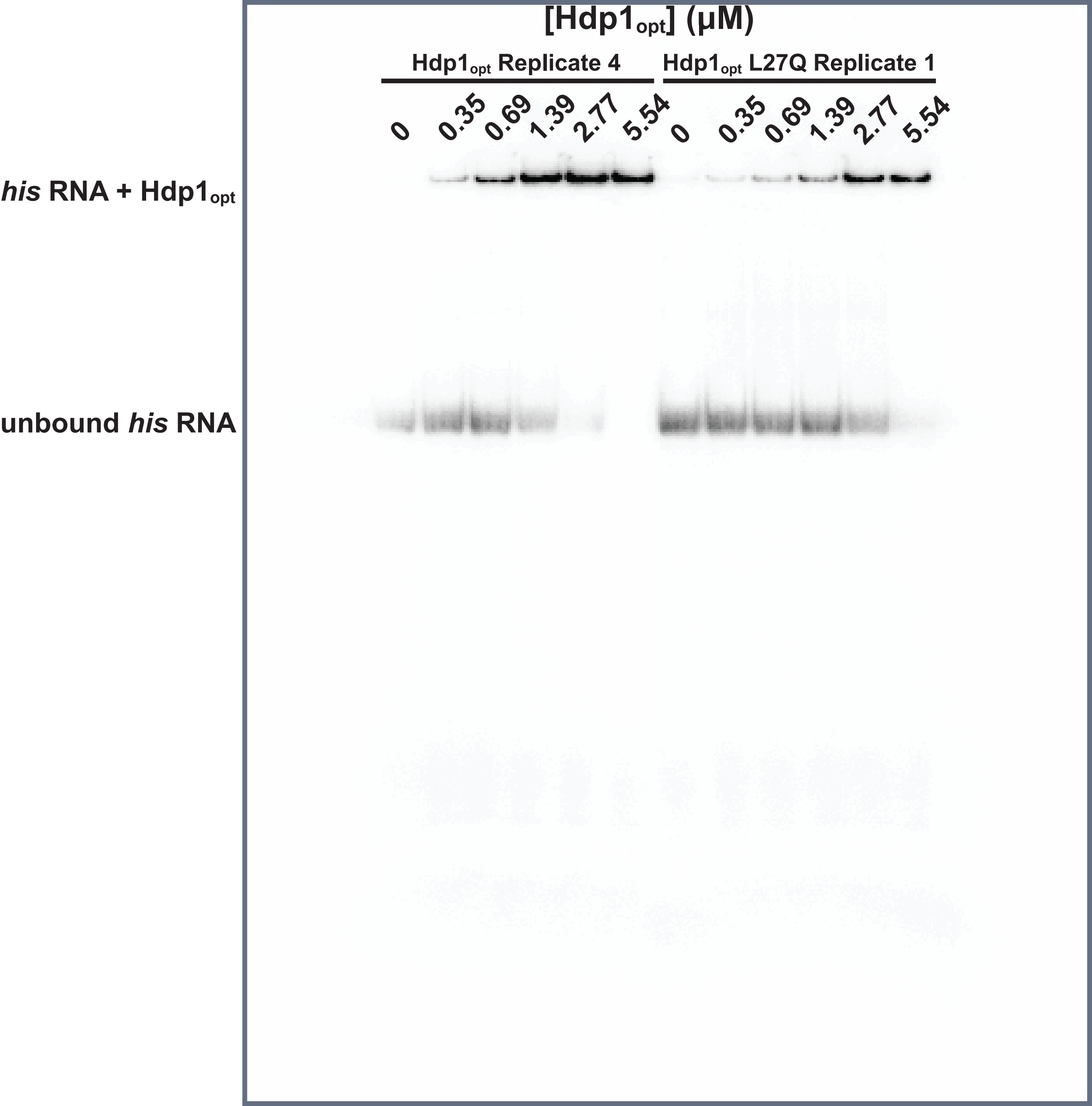
[Hdp1opt] (μM)
Hdp1opt Replicate 4
Hdp1opt L27Q Replicate 1
0
0.35
0.69
1.39
2.77
5.54
0
0.35
0.69
1.39
2.77
5.54
his RNA + Hdp1opt
unbound his RNA
