# Supplementary material for: Rescue of Escherichia coli auxotrophy by de novo small proteins
Source: eLife. 2023 Mar 15;12:e78299. doi: 10.7554/eLife.78299 (PMC10065794; doi:10.7554/eLife.78299)
Supplement: Figure 3—source data 1. [file elife-78299-fig3-data1.zip › Figure 3D - labeled source data 1.pdf]

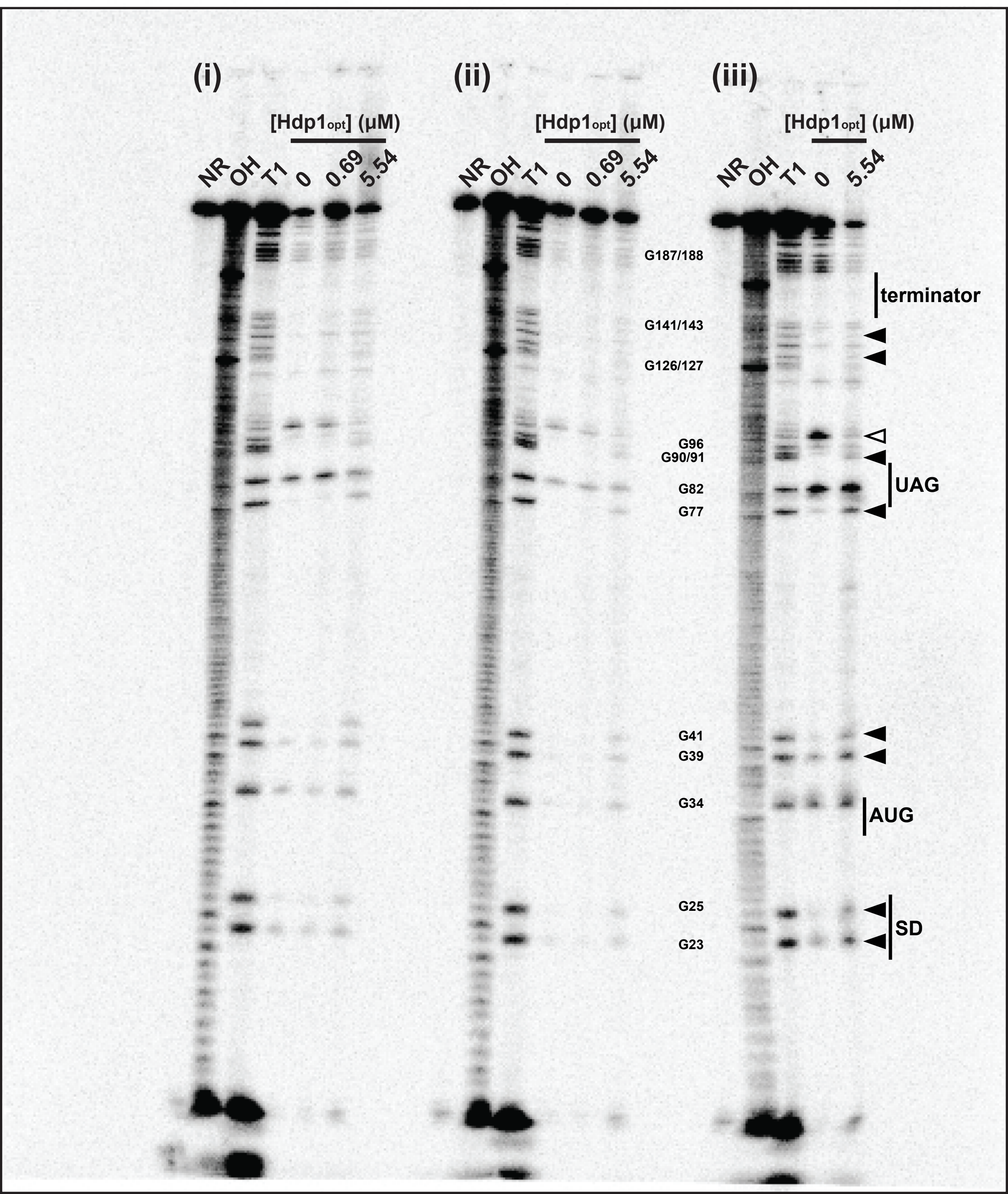

(i)
[Hdp1opt] (μM)
NR
OH
T1
0
0.69
5.54
(ii)
[Hdp1opt] (μM)
NR
OH
T1
0
0.69
5.54
(iii)
[Hdp1opt] (μM)
NR
OH
T1
0
5.54
G187/188
terminator
G141/143
G126/127
G96
G90/91
G82
UAG
G77
G41
G39
G34
AUG
G25
SD
G23
