# Supplementary figures and images for: Rescue of Escherichia coli auxotrophy by de novo small proteins
Source: eLife. 2023 Mar 15;12:e78299. doi: 10.7554/eLife.78299 (PMC10065794; doi:10.7554/eLife.78299)

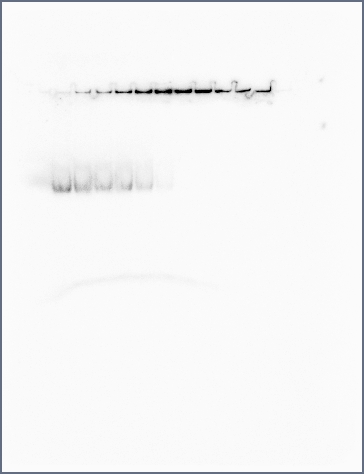

Supplement: Figure 3—source data 1. [file elife-78299-fig3-data1.zip › Figure 3B-source data 1.jpeg]

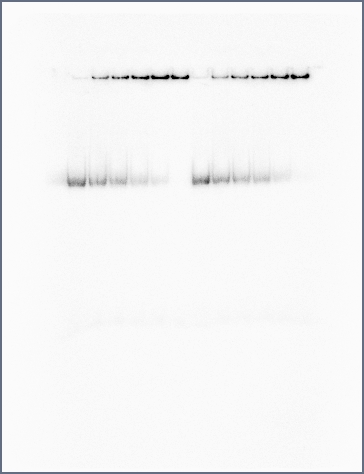

Supplement: Figure 3—source data 1. [file elife-78299-fig3-data1.zip › Figure 3B-source data 2.jpeg]

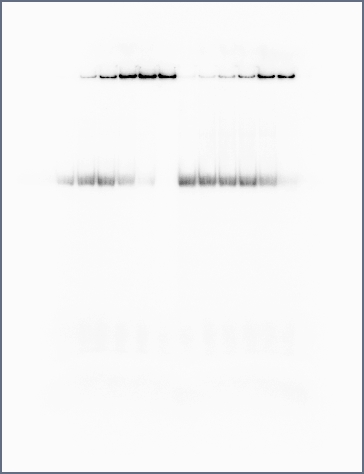

Supplement: Figure 3—source data 1. [file elife-78299-fig3-data1.zip › Figure 3B-source data 3.jpeg]

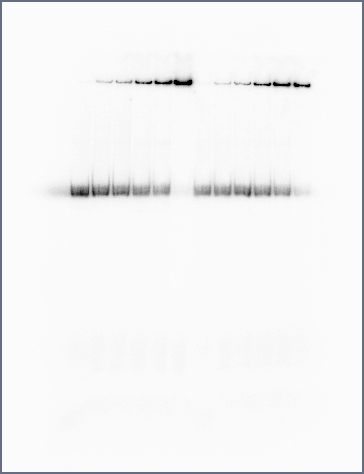

Supplement: Figure 3—source data 1. [file elife-78299-fig3-data1.zip › Figure 3B-source data 4.jpeg]

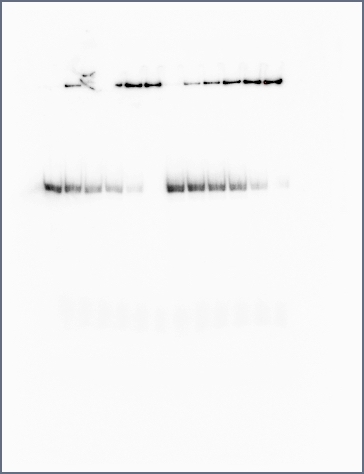

Supplement: Figure 3—source data 1. [file elife-78299-fig3-data1.zip › Figure 3B-source data 5.jpeg]

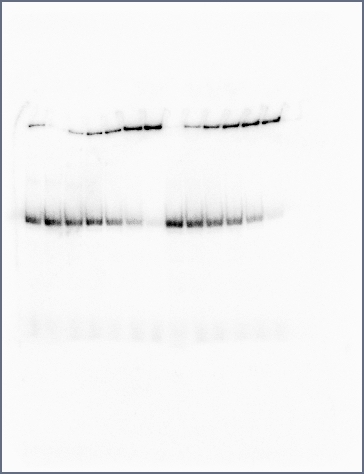

Supplement: Figure 3—source data 1. [file elife-78299-fig3-data1.zip › Figure 3B-source data 6.jpeg]

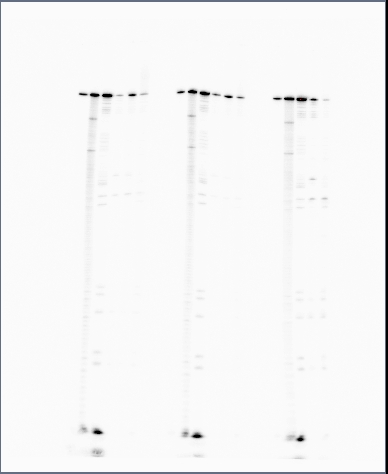

Supplement: Figure 3—source data 1. [file elife-78299-fig3-data1.zip › Figure 3D-source data 1.jpeg]

*his* RNA + Hdp1<sub>opt</sub>

unbound *his* RNA

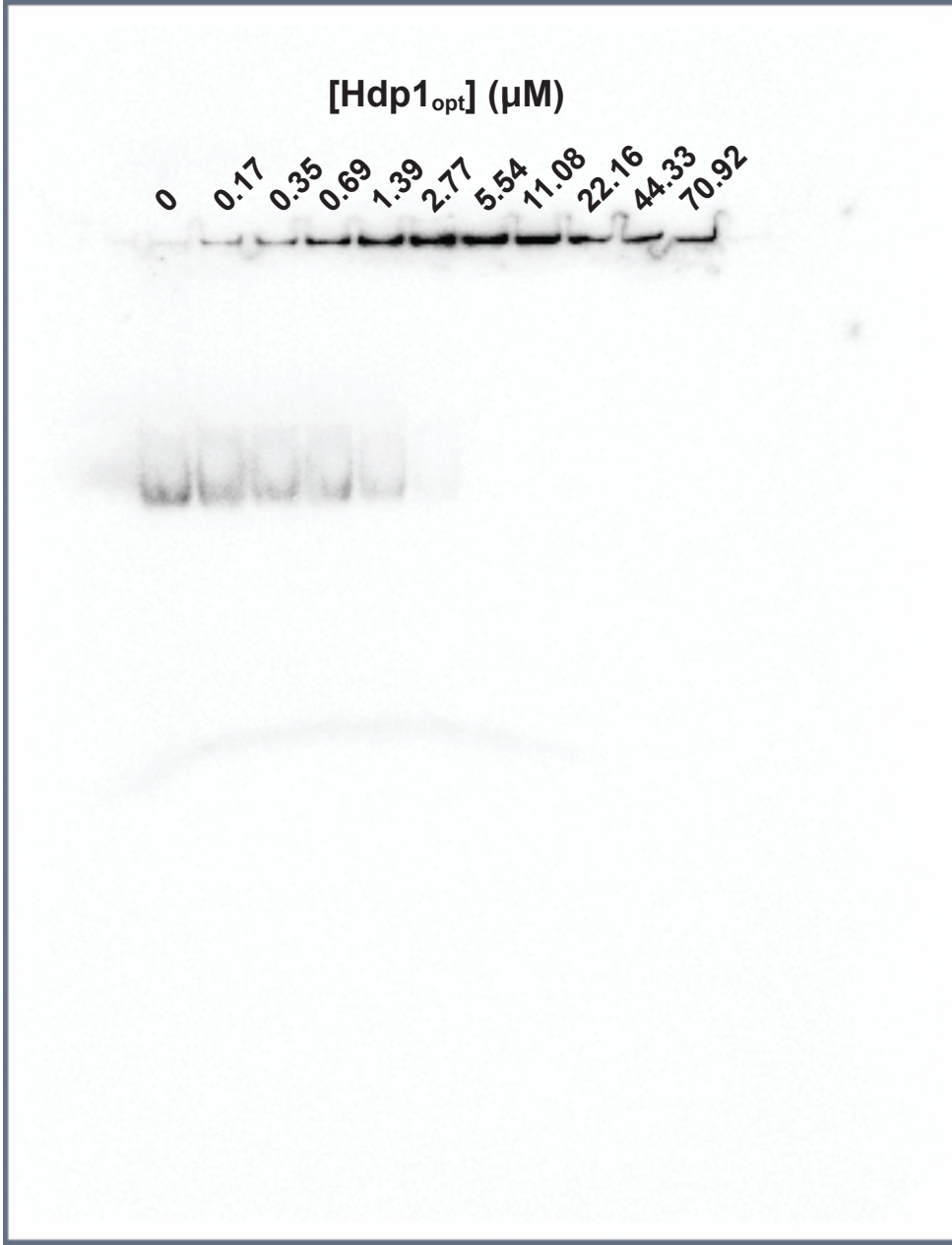

Supplement: Figure 3—source data 1. [file elife-78299-fig3-data1.zip › Figure 3B - labeled source data 1.pdf]

*his* RNA + Hdp1<sub>opt</sub>

unbound *his* RNA

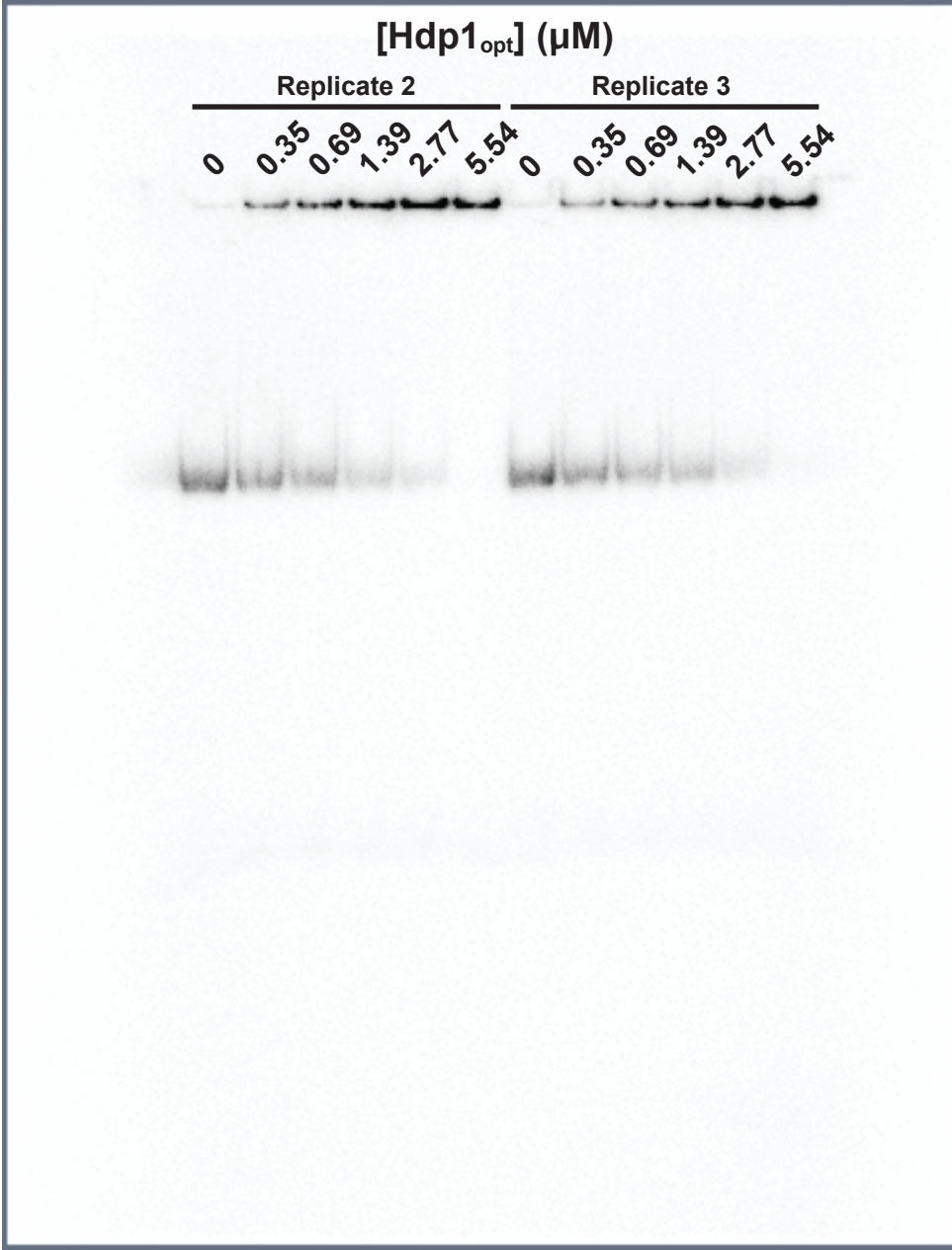

Supplement: Figure 3—source data 1. [file elife-78299-fig3-data1.zip › Figure 3B - labeled source data 2.pdf]

*his* RNA + Hdp1<sub>opt</sub>

unbound *his* RNA

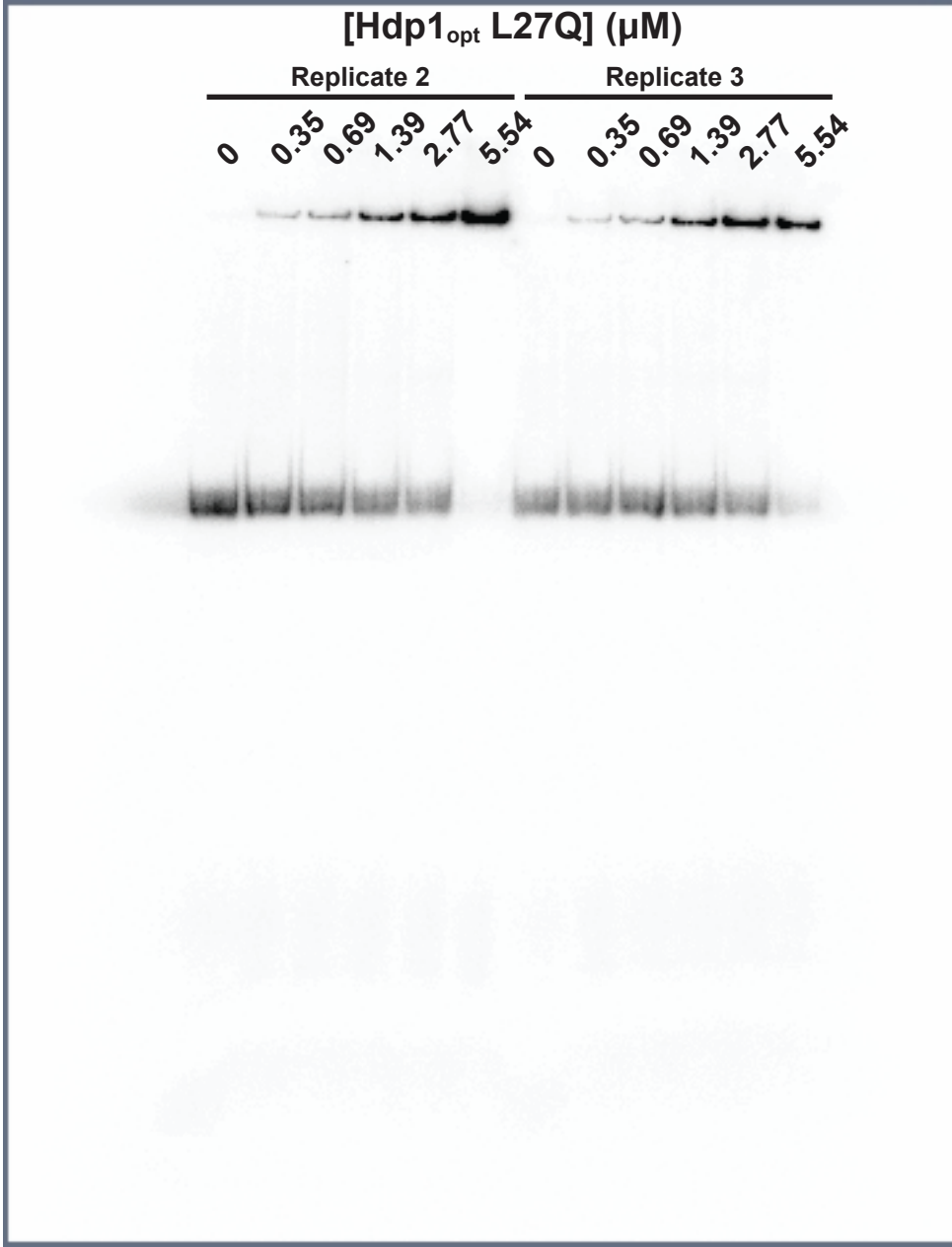

Supplement: Figure 3—source data 1. [file elife-78299-fig3-data1.zip › Figure 3B - labeled source data 4.pdf]

*thr* RNA + Hdp1<sub>opt</sub>

unbound *thr* RNA

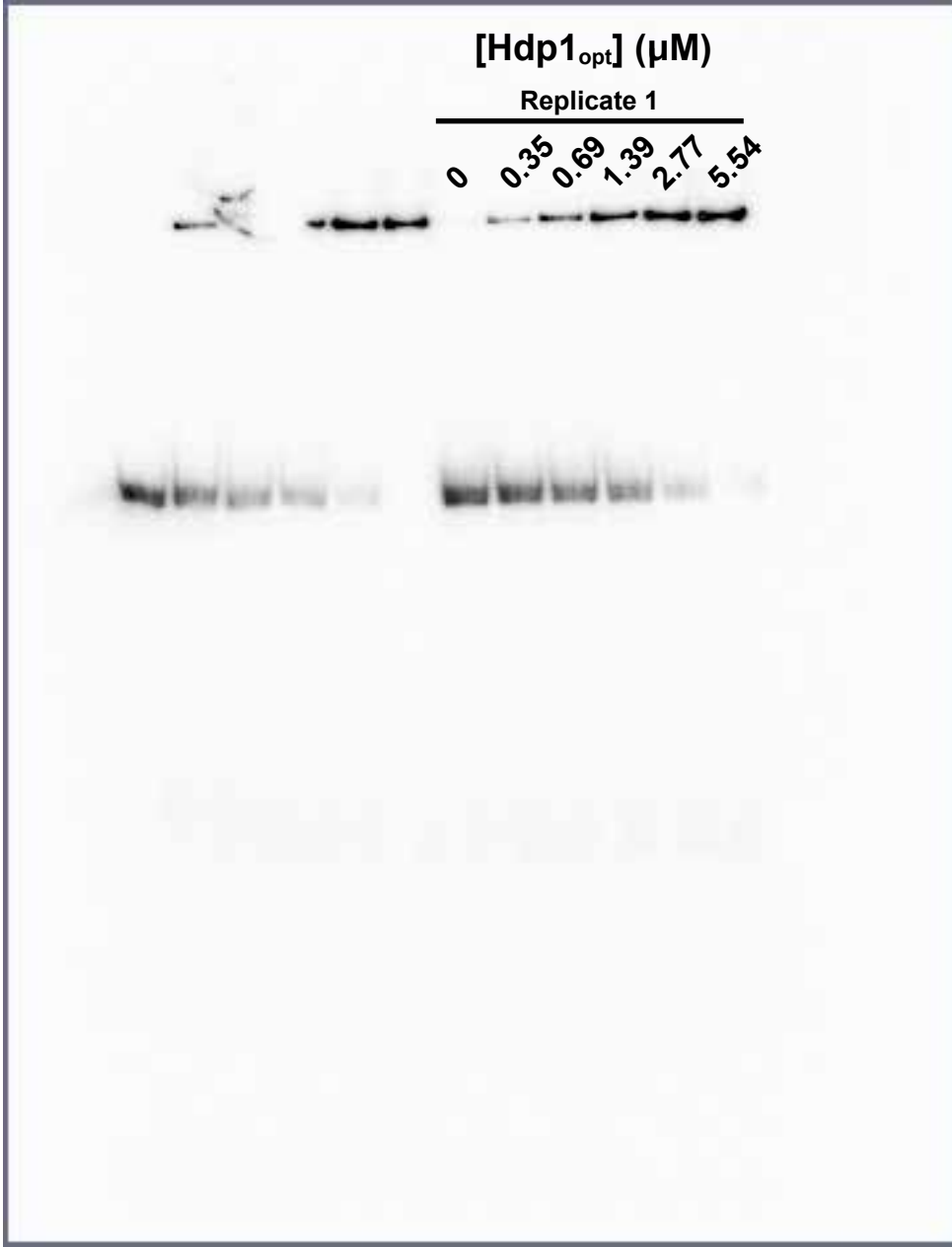

Supplement: Figure 3—source data 1. [file elife-78299-fig3-data1.zip › Figure 3B - labeled source data 5.pdf]

*thr* RNA + Hdp1<sub>opt</sub>

unbound *thr* RNA

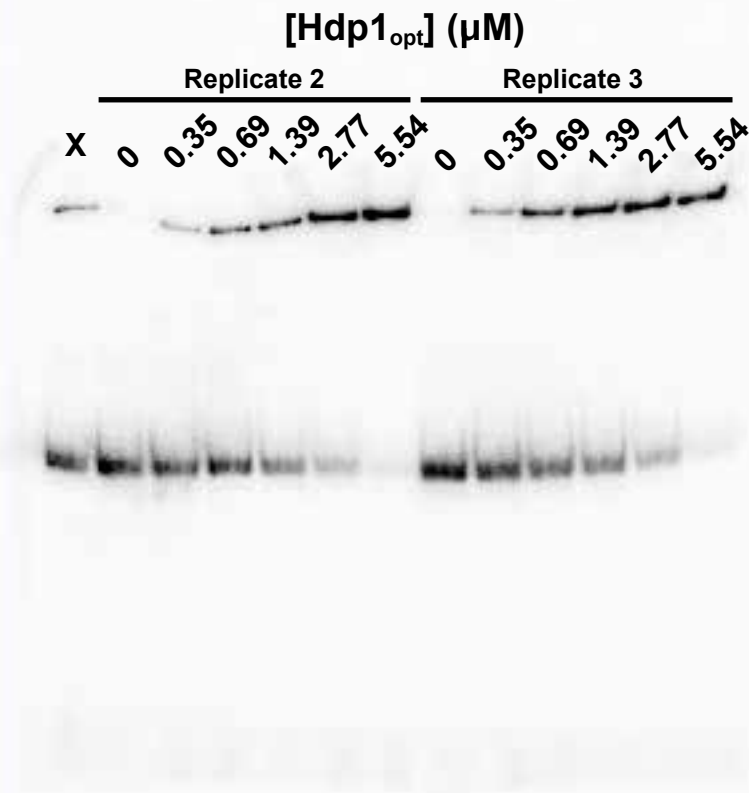

Supplement: Figure 3—source data 1. [file elife-78299-fig3-data1.zip › Figure 3B - labeled source data 6.pdf]

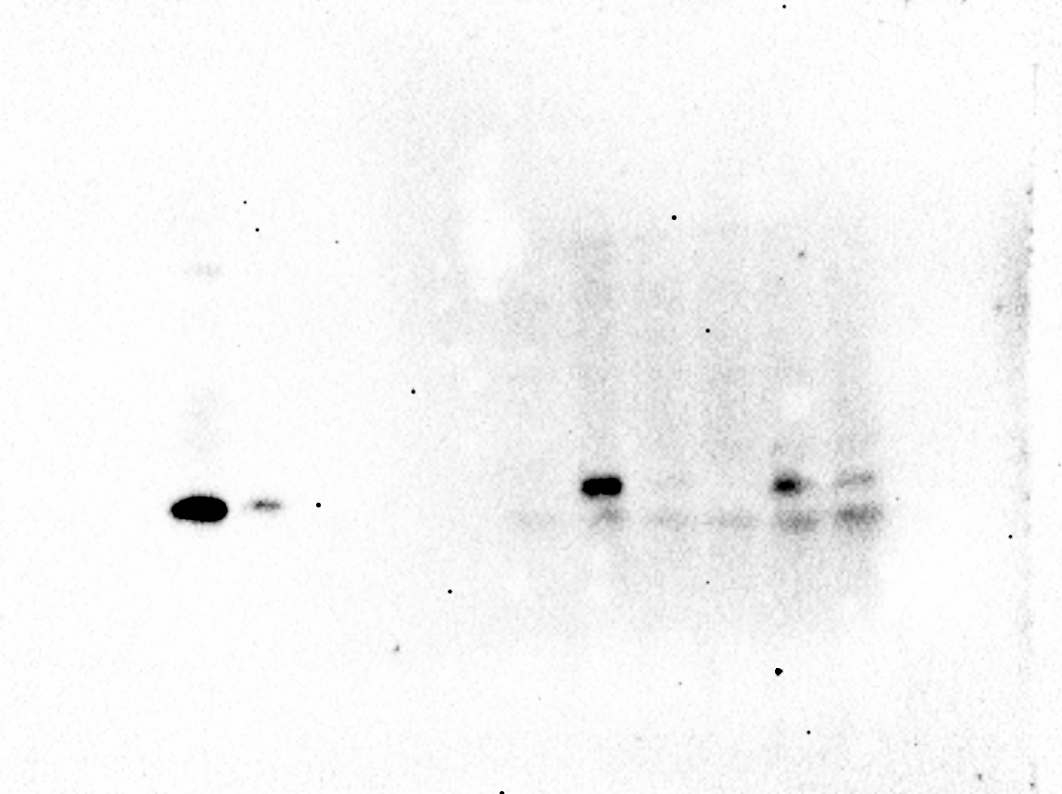

Supplement: Figure 3—figure supplement 2—source data 1. — HA-tagged proteins of interested were detected using HRP-conjugated anti-HA mouse monoclonal antibody and Amersham ECL Prime Western Blotting Detection Reagent (Cytiva) and visualized using a Bio-Rad ChemiDoc MP System (Chemi Hi Sensitivity setting). Uncropped membrane from experimental replicate 1. [file elife-78299-fig3-figsupp2-data1.zip › Figure 3-figure supplement 2-source data 1.tiff]

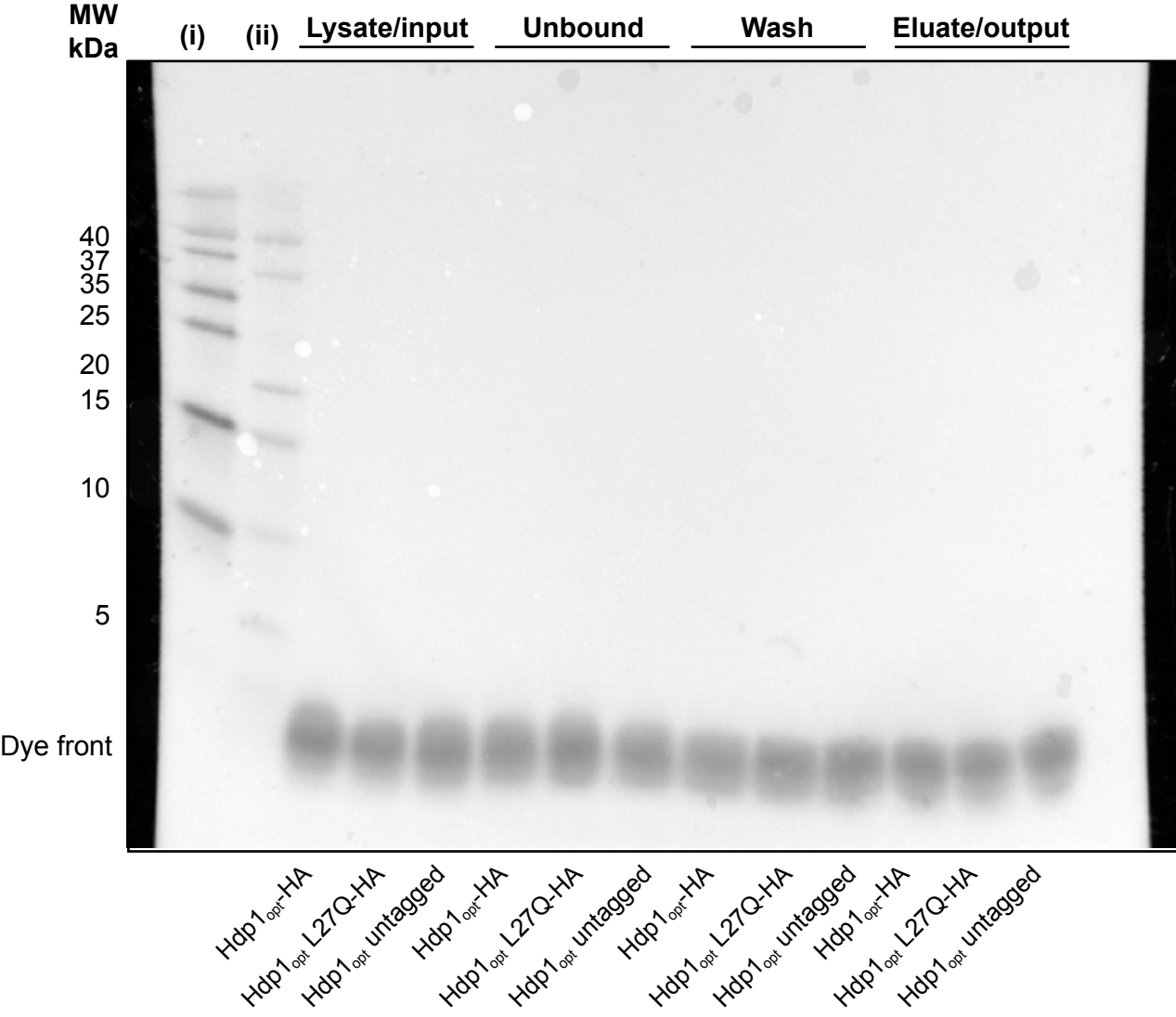

Supplement: Figure 3—figure supplement 2—source data 2. — White light image of membrane to show protein ladders for size reference. Uncropped membrane from experimental replicate 1. [file elife-78299-fig3-figsupp2-data2.zip › Figure 3-figure supplement 2 - labeled source data 2.pdf]

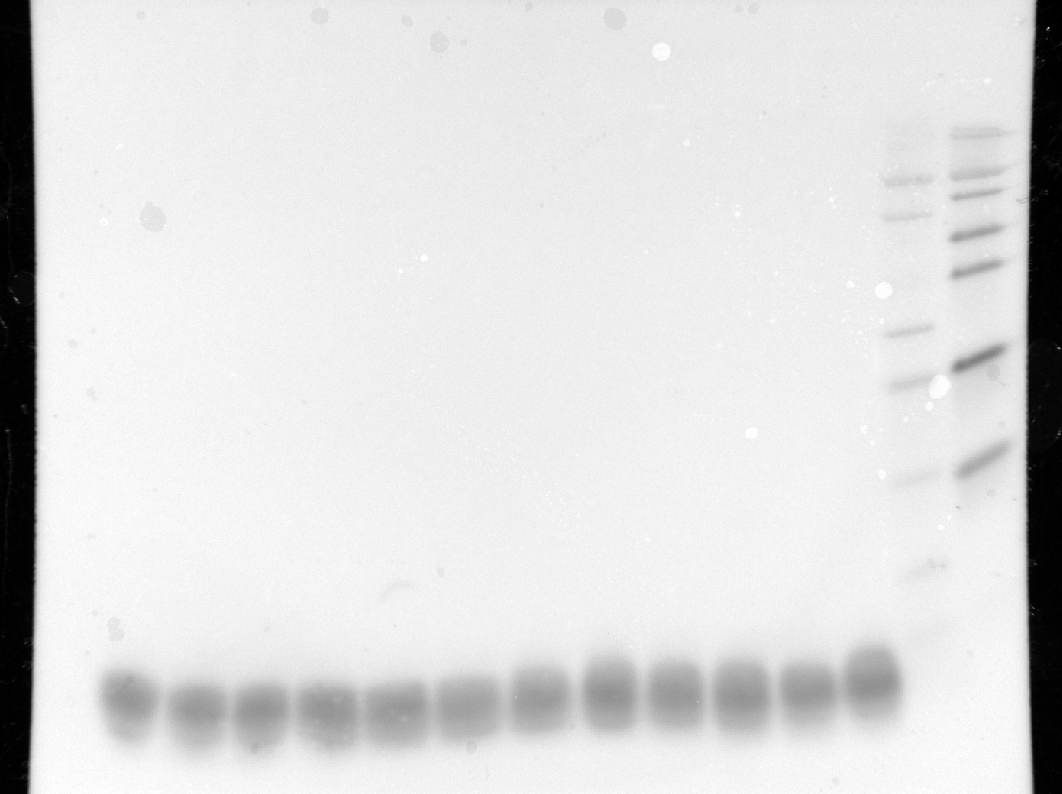

Supplement: Figure 3—figure supplement 2—source data 2. — White light image of membrane to show protein ladders for size reference. Uncropped membrane from experimental replicate 1. [file elife-78299-fig3-figsupp2-data2.zip › Figure 3-figure supplement 2-source data 2.tiff]

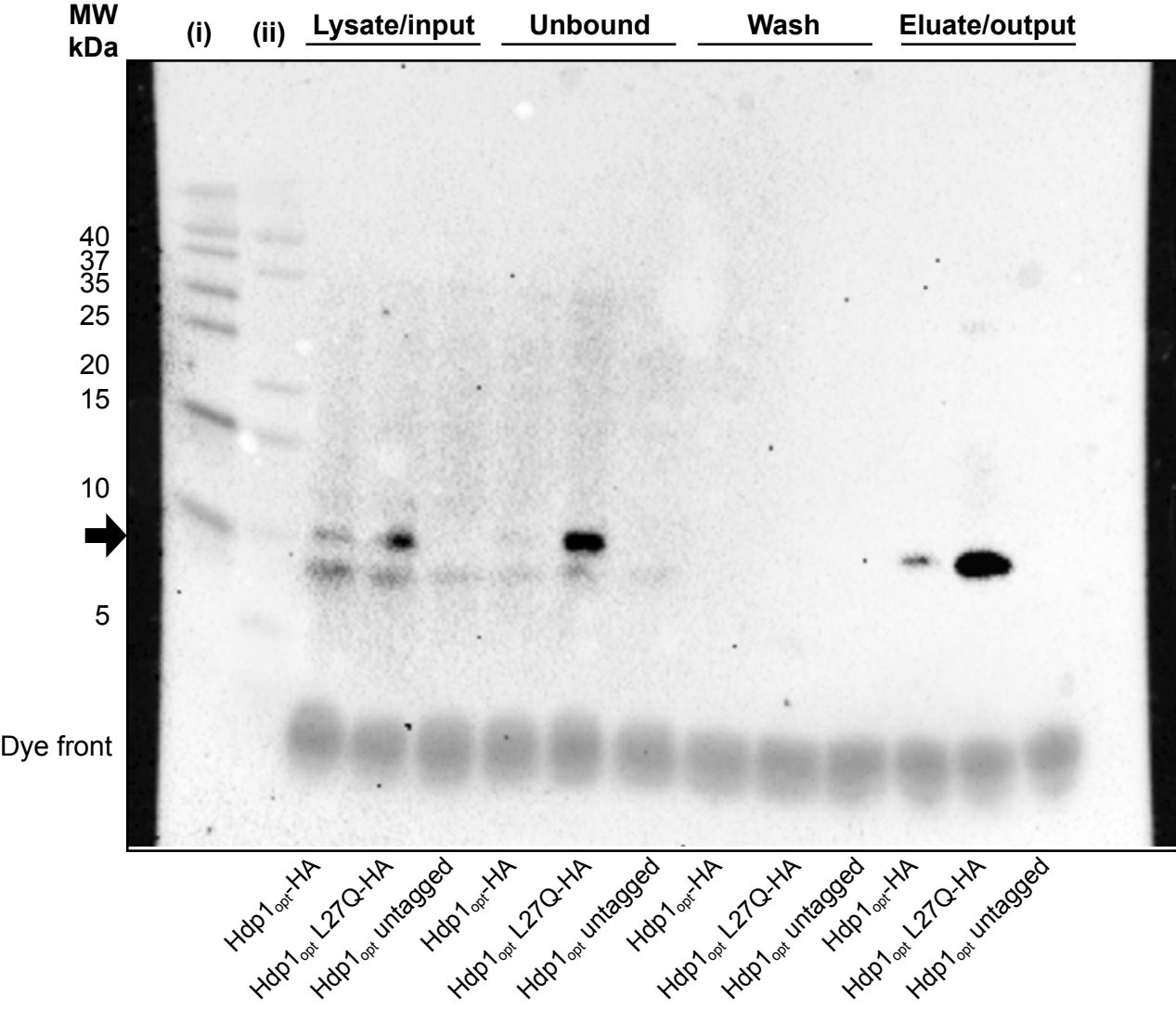

Supplement: Figure 3—figure supplement 2—source data 3. — Merged image of membranes from Figure 3—figure supplement 2—source data 1 and Figure 3—figure supplement 2—source data 2 to show protein ladders for size reference alongside detected proteins of interest. Uncropped membrane from experimental replicate 1; included in Figure 3—figure supplement 2 as a representative western blot for the pull-down assays. [file elife-78299-fig3-figsupp2-data3.zip › Figure 3-figure supplement 2 - labeled source data 3.pdf]

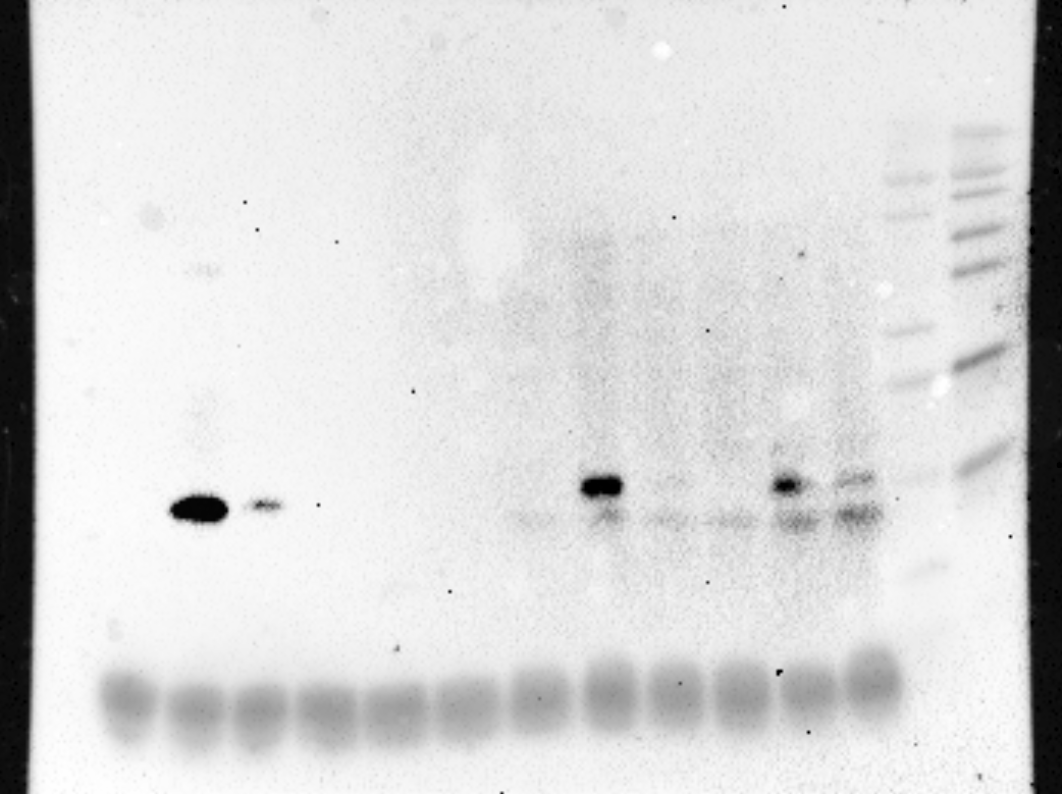

Supplement: Figure 3—figure supplement 2—source data 3. — Merged image of membranes from Figure 3—figure supplement 2—source data 1 and Figure 3—figure supplement 2—source data 2 to show protein ladders for size reference alongside detected proteins of interest. Uncropped membrane from experimental replicate 1; included in Figure 3—figure supplement 2 as a representative western blot for the pull-down assays. [file elife-78299-fig3-figsupp2-data3.zip › Figure 3-figure supplement 2-source data 3.tiff]

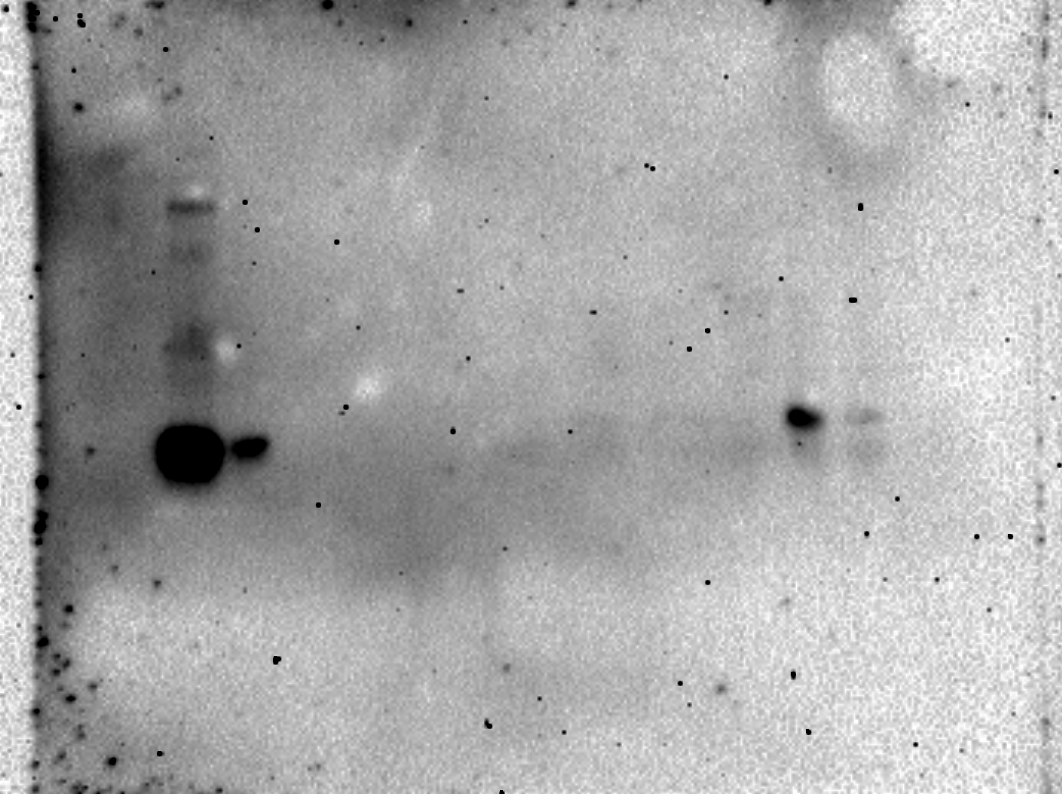

Supplement: Figure 3—figure supplement 2—source data 4. — HA-tagged proteins of interested were detected using HRP-conjugated anti-HA mouse monoclonal antibody and Amersham ECL Prime Western Blotting Detection Reagent (Cytiva) and visualized using a Bio-Rad ChemiDoc MP System (Chemi Hi Sensitivity setting). Uncropped membrane from experimental replicate 2. [file elife-78299-fig3-figsupp2-data4.zip › Figure 3-figure supplement 2-source data 4.tiff]

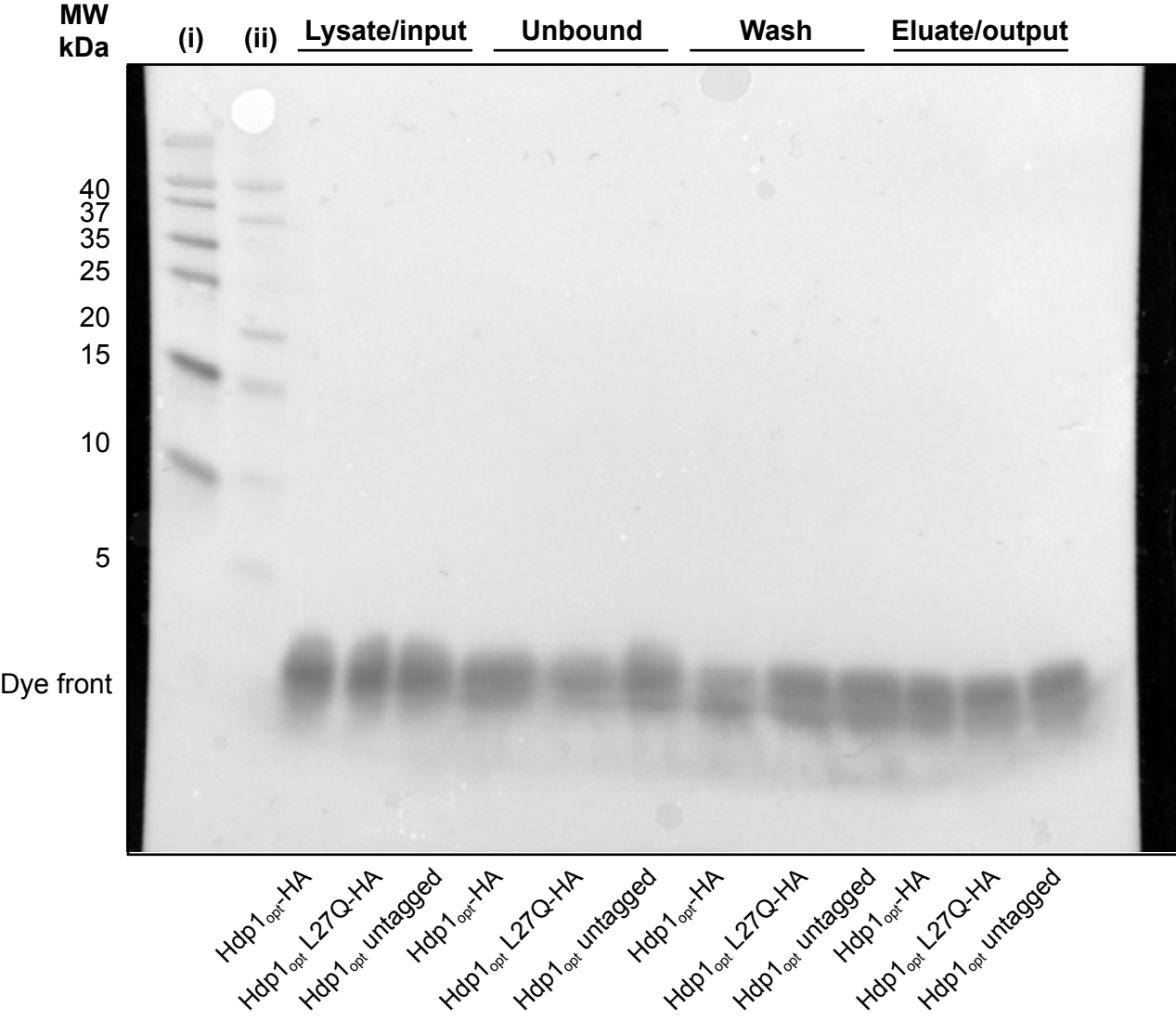

Supplement: Figure 3—figure supplement 2—source data 5. — White light image of membrane to show protein ladders for size reference. Uncropped membrane from experimental replicate 2. [file elife-78299-fig3-figsupp2-data5.zip › Figure 3-figure supplement 2 - labeled source data 5.pdf]

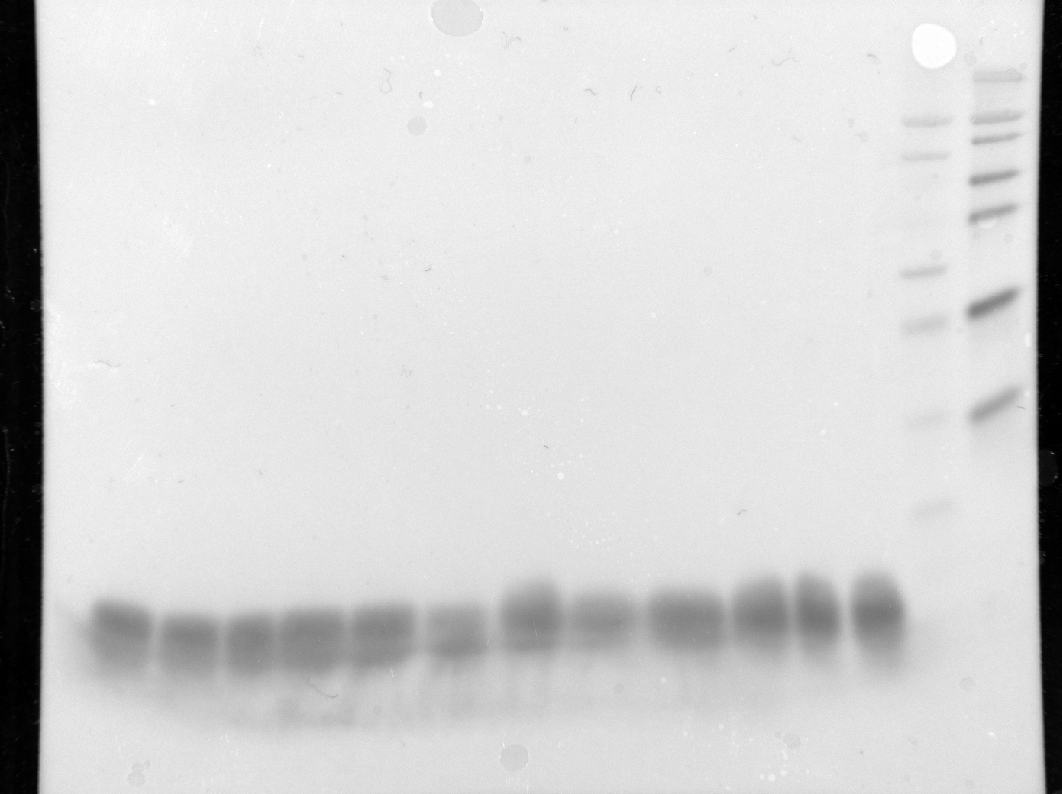

Supplement: Figure 3—figure supplement 2—source data 5. — White light image of membrane to show protein ladders for size reference. Uncropped membrane from experimental replicate 2. [file elife-78299-fig3-figsupp2-data5.zip › Figure 3-figure supplement 2-source data 5.tiff]

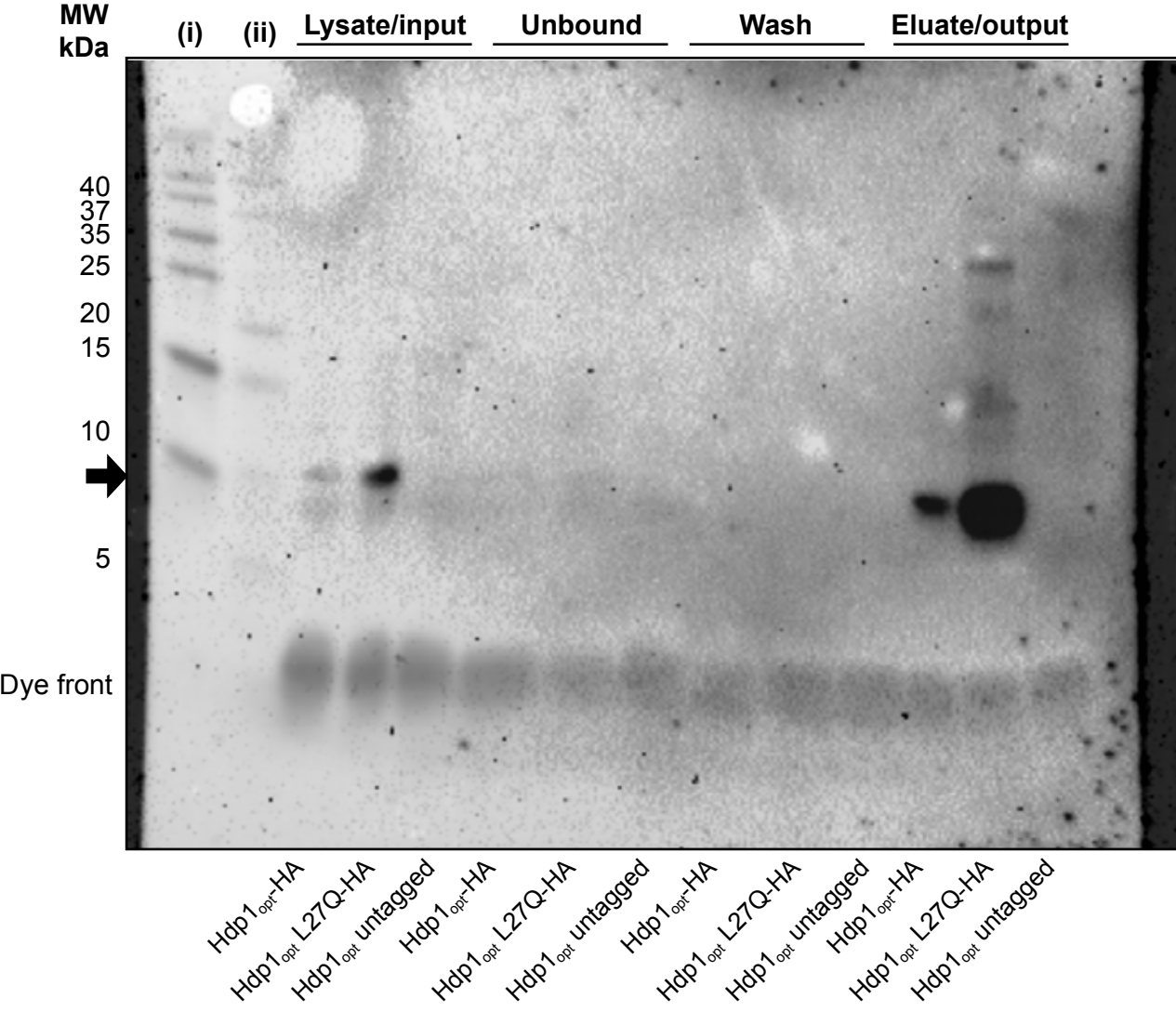

Supplement: Figure 3—figure supplement 2—source data 6. — Merged image of membranes from Figure 3—figure supplement 2—source data 4 and Figure 3—figure supplement 2—source data 5 to show protein ladders for size reference alongside detected proteins of interest. Uncropped membrane from experimental replicate 2. [file elife-78299-fig3-figsupp2-data6.zip › Figure 3-figure supplement 2 - labeled source data 6.pdf]

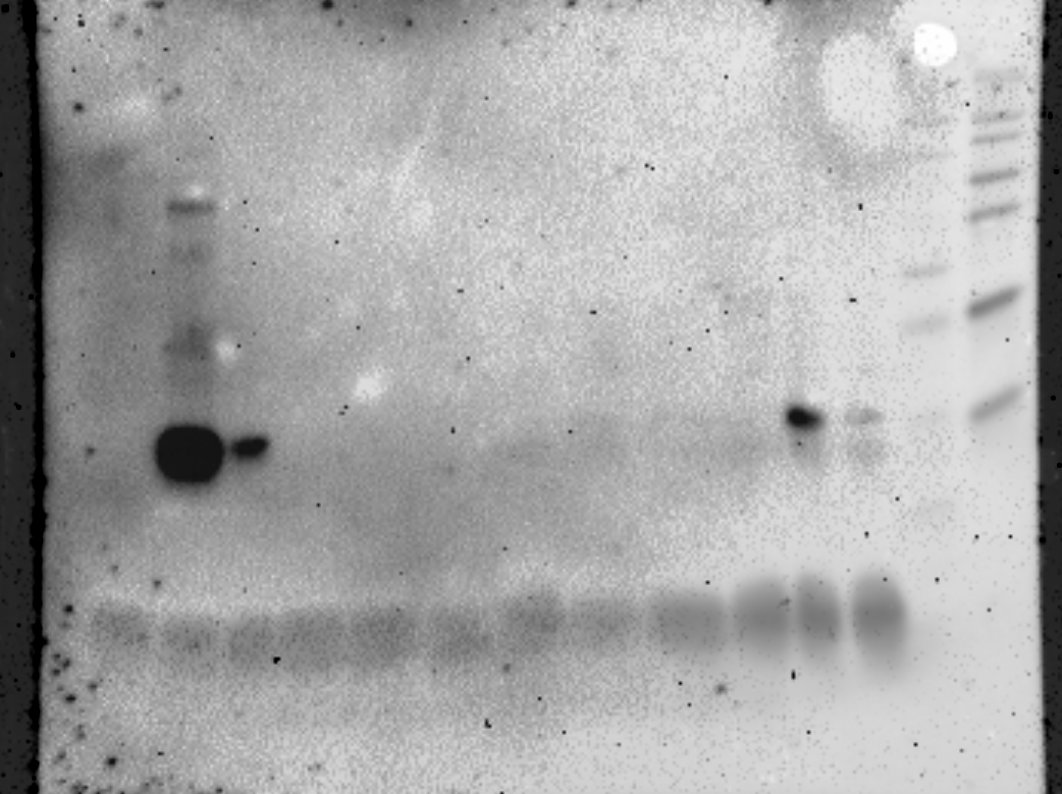

Supplement: Figure 3—figure supplement 2—source data 6. — Merged image of membranes from Figure 3—figure supplement 2—source data 4 and Figure 3—figure supplement 2—source data 5 to show protein ladders for size reference alongside detected proteins of interest. Uncropped membrane from experimental replicate 2. [file elife-78299-fig3-figsupp2-data6.zip › Figure 3-figure supplement 2-source data 6.tiff]

*his* RNA + Hdp1<sub>opt</sub>

unbound *his* RNA

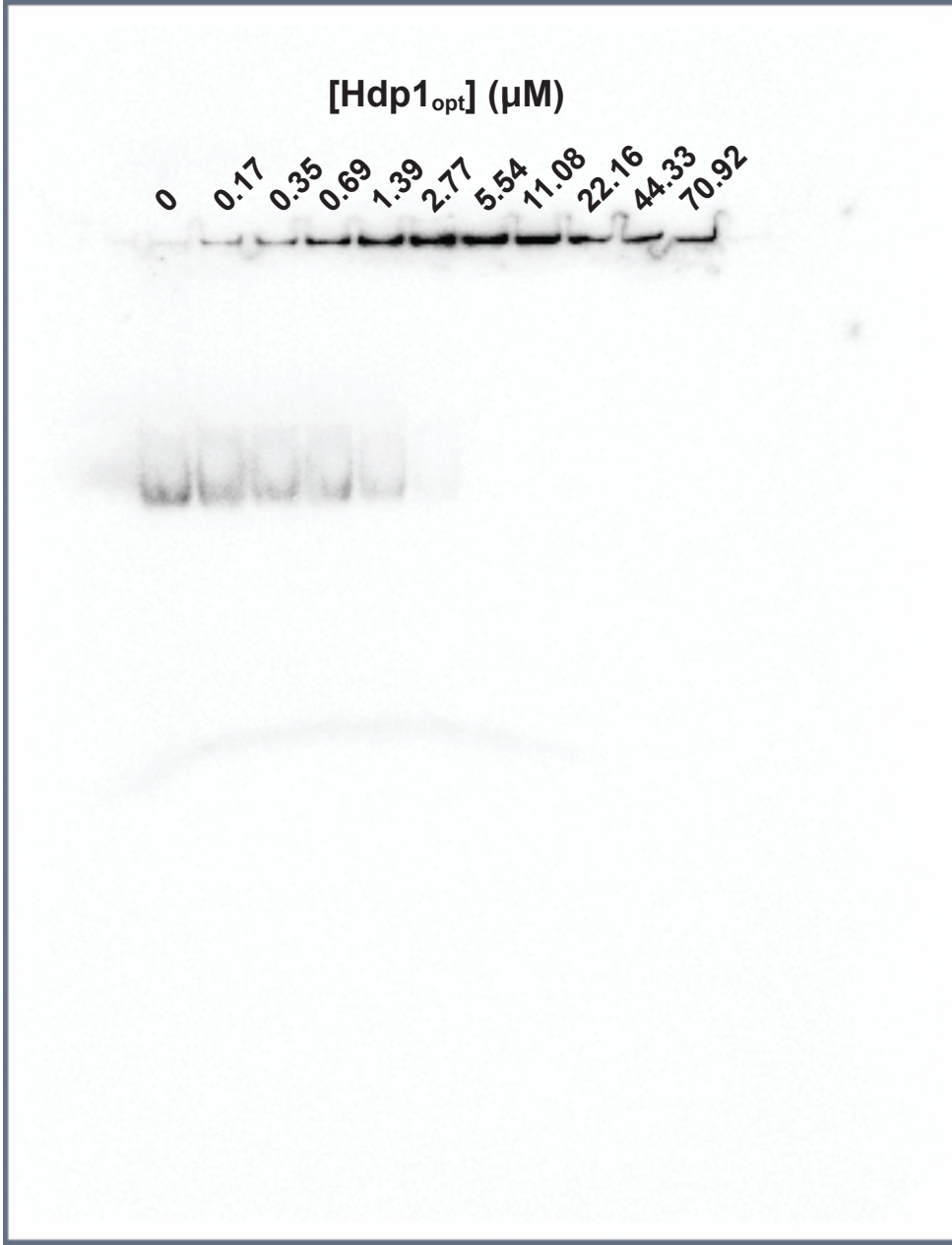

Supplement: Figure 3—figure supplement 3—source data 1. — Uncropped gel from experimental replicate 1. [file elife-78299-fig3-figsupp3-data1.zip › Figure 3-figure supplement 3 - labeled source data 1.pdf]

*his* RNA + Hdp1<sub>opt</sub>

unbound *his* RNA

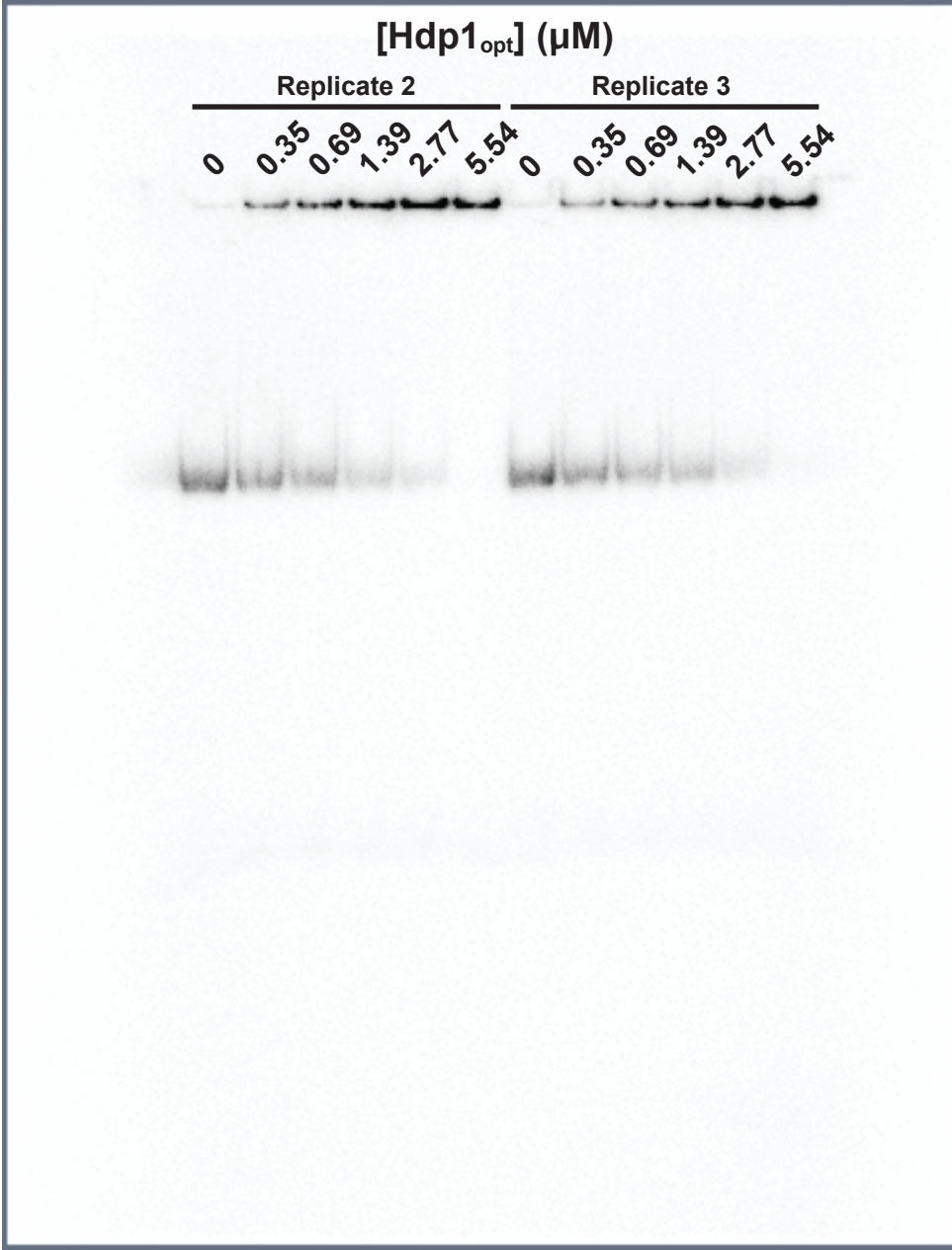

Supplement: Figure 3—figure supplement 3—source data 2. — Uncropped gel from experimental replicates 2 and 3. Experimental replicate 2 is included in the main text figure as a representative EMSA gel image. [file elife-78299-fig3-figsupp3-data2.zip › Figure 3-figure supplement 3 - labeled source data 2.pdf]

*his* RNA + Hdp1<sub>opt</sub>

unbound *his* RNA

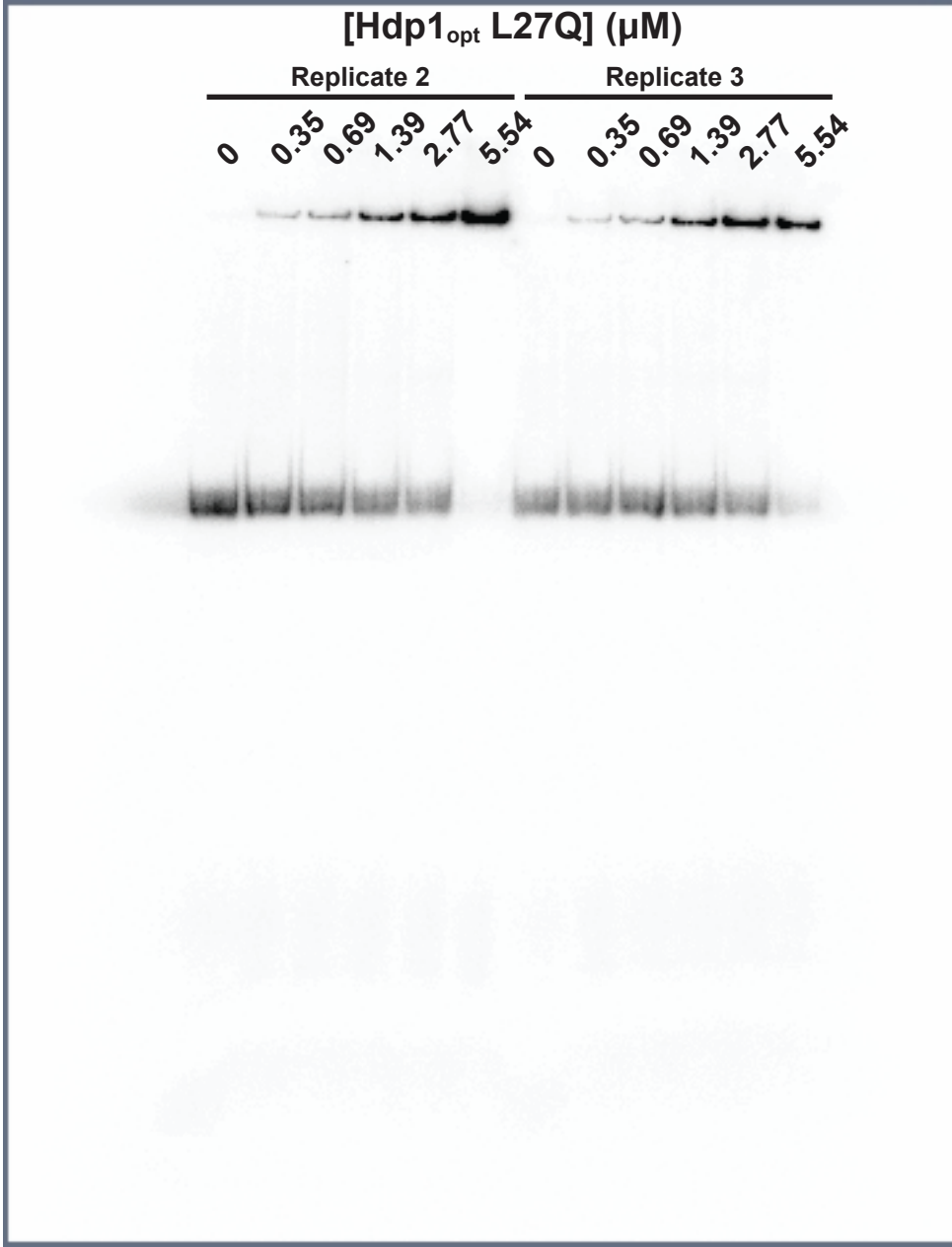

Supplement: Figure 3—figure supplement 3—source data 4. — Uncropped gel from experimental replicates 2 and 3. [file elife-78299-fig3-figsupp3-data4.zip › Figure 3-figure supplement 3 - labeled source data 4.pdf]

*thr* RNA + Hdp1<sub>opt</sub>

unbound *thr* RNA

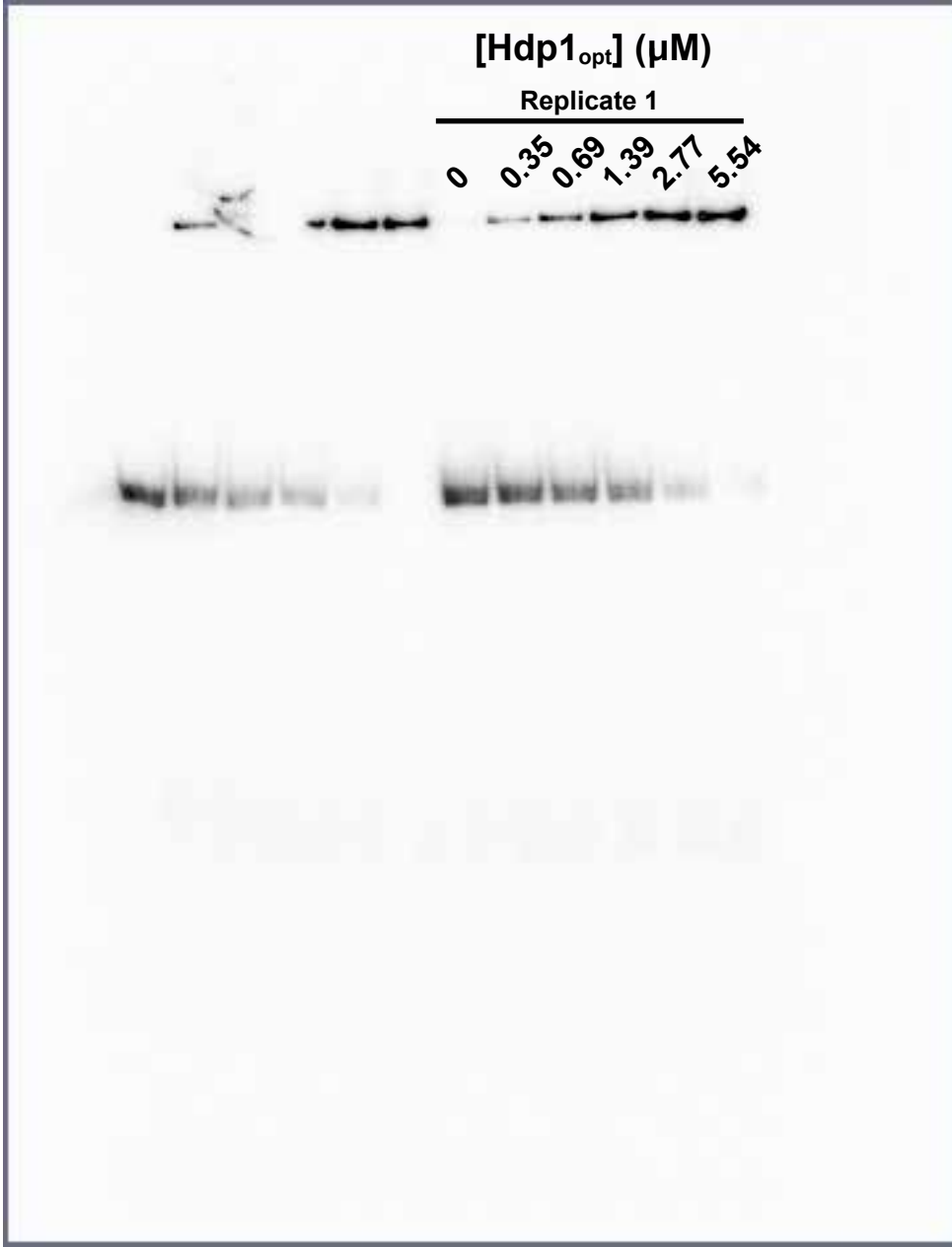

Supplement: Figure 3—figure supplement 3—source data 5. — Uncropped gel from experimental replicate 1; included in Figure 3—figure supplement 3 as a representative EMSA gel image. Unlabeled experiment on the left of the gel was not used for quantification or analysis due to the gel ripping. [file elife-78299-fig3-figsupp3-data5.zip › Figure 3-figure supplement 3 - labeled source data 5.pdf]

*thr* RNA + Hdp1<sub>opt</sub>

unbound *thr* RNA

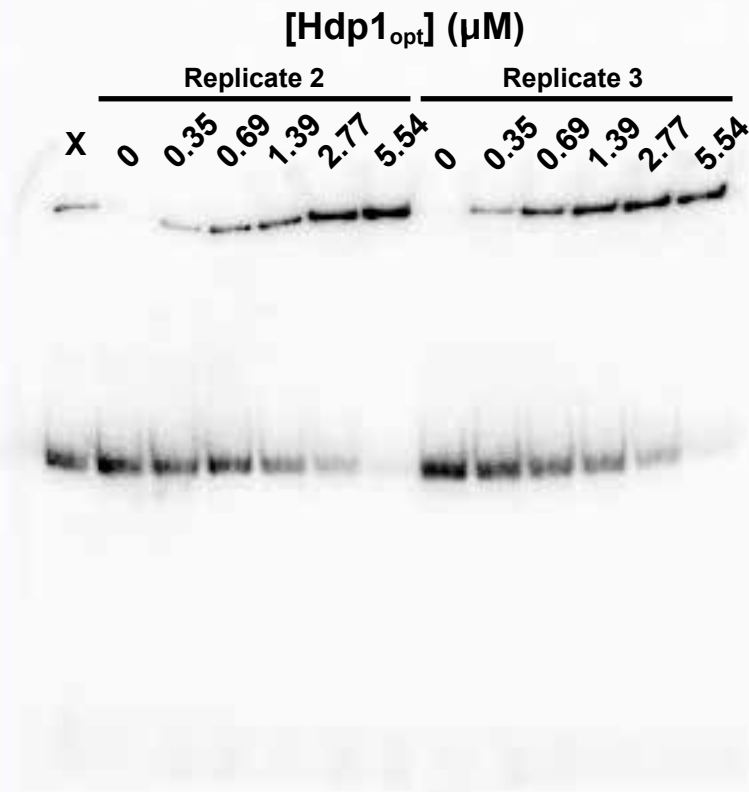

Supplement: Figure 3—figure supplement 3—source data 6. — Uncropped gel from experimental replicates 2 and 3. ‘X’ denotes a well in which a reaction was loaded in the incorrect order. [file elife-78299-fig3-figsupp3-data6.zip › Figure 3-figure supplement 3 - labeled source data 6.pdf]

(i)

[Hdp1<sub>opt</sub>] (μM)  
NR OH T1 0 0.69 5.54

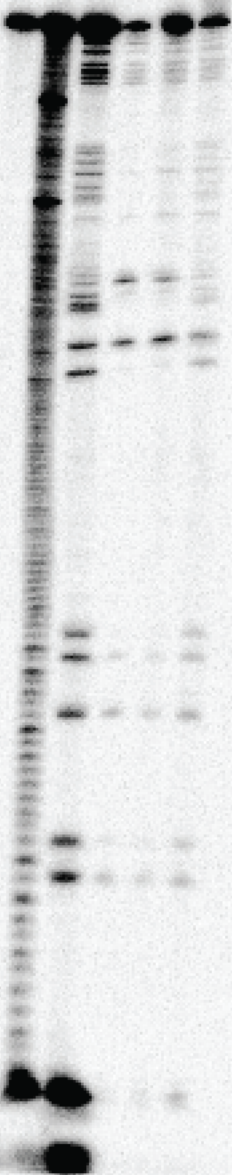

(ii)

[Hdp1<sub>opt</sub>] (μM)  
NR OH T1 0 0.69 5.54

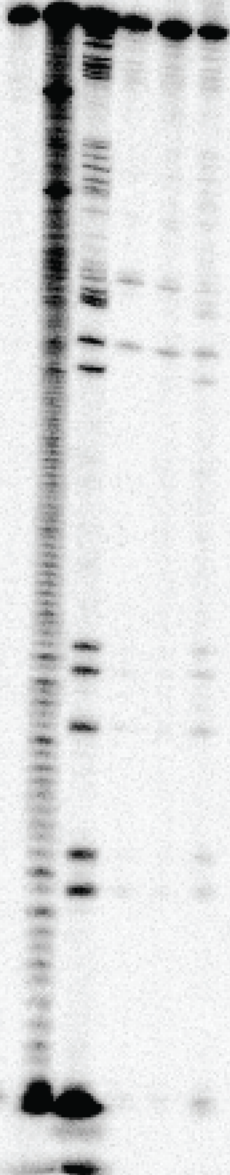

(iii)

[Hdp1<sub>opt</sub>] (μM)  
NR OH T1 0 5.54

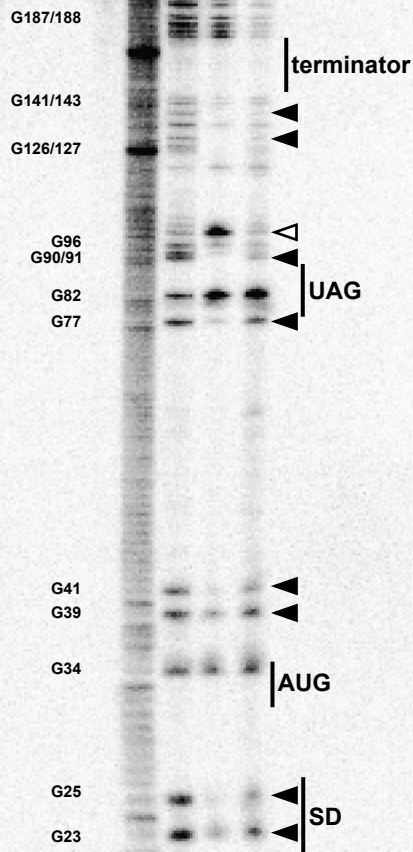

Supplement: Figure 3—figure supplement 4—source data 1. — NR denotes RNA subject to no reaction, OH indicates partial alkaline hydrolysis, and T1 is an RNase T1 digest of the RNA under denaturing conditions used to map the RNA sequence. (i) Probing reactions incubated with 0.05U T1 RNase for 5 min. (ii) Probing reactions incubated with 0.01U T1 RNase for 5 min. (iii) Probing reactions incubated with 0.05U T1 RNase for 10 min. Numbering of G nucleotides is shown on the left. Arrows highlight changes in RNA cleavage in the presence of Hdp1opt: black arrows indicate nucleotides with increased cleavage and white arrows indicate reduced cleavage. Sequence and/or structure characteristics of the his operator RNA are also indicated on the right (i.e., the Shine-Dalgarno sequence [SD], start codon [AUG], and stop codon [UAG] of the hisL leader peptide coding sequence). The reactions from (iii) are included in the main text figure as a representative T1 RNase probing gel image; all replicates are in included in Figure 3—figure supplement 4. All reaction sets were performed with independent RNA and protein dilutions. [file elife-78299-fig3-figsupp4-data1.zip › Figure 3-figure supplement 4 - labeled source data 1.pdf]
