# Supplementary material for: Rescue of Escherichia coli auxotrophy by de novo small proteins
Source: eLife. 2023 Mar 15;12:e78299. doi: 10.7554/eLife.78299 (PMC10065794; doi:10.7554/eLife.78299)
Supplement: Figure 3—figure supplement 2—source data 4. — HA-tagged proteins of interested were detected using HRP-conjugated anti-HA mouse monoclonal antibody and Amersham ECL Prime Western Blotting Detection Reagent (Cytiva) and visualized using a Bio-Rad ChemiDoc MP System (Chemi Hi Sensitivity setting). Uncropped membrane from experimental replicate 2. [file elife-78299-fig3-figsupp2-data4.zip › Figure 3-figure supplement 2 - labeled source data 4.pdf]

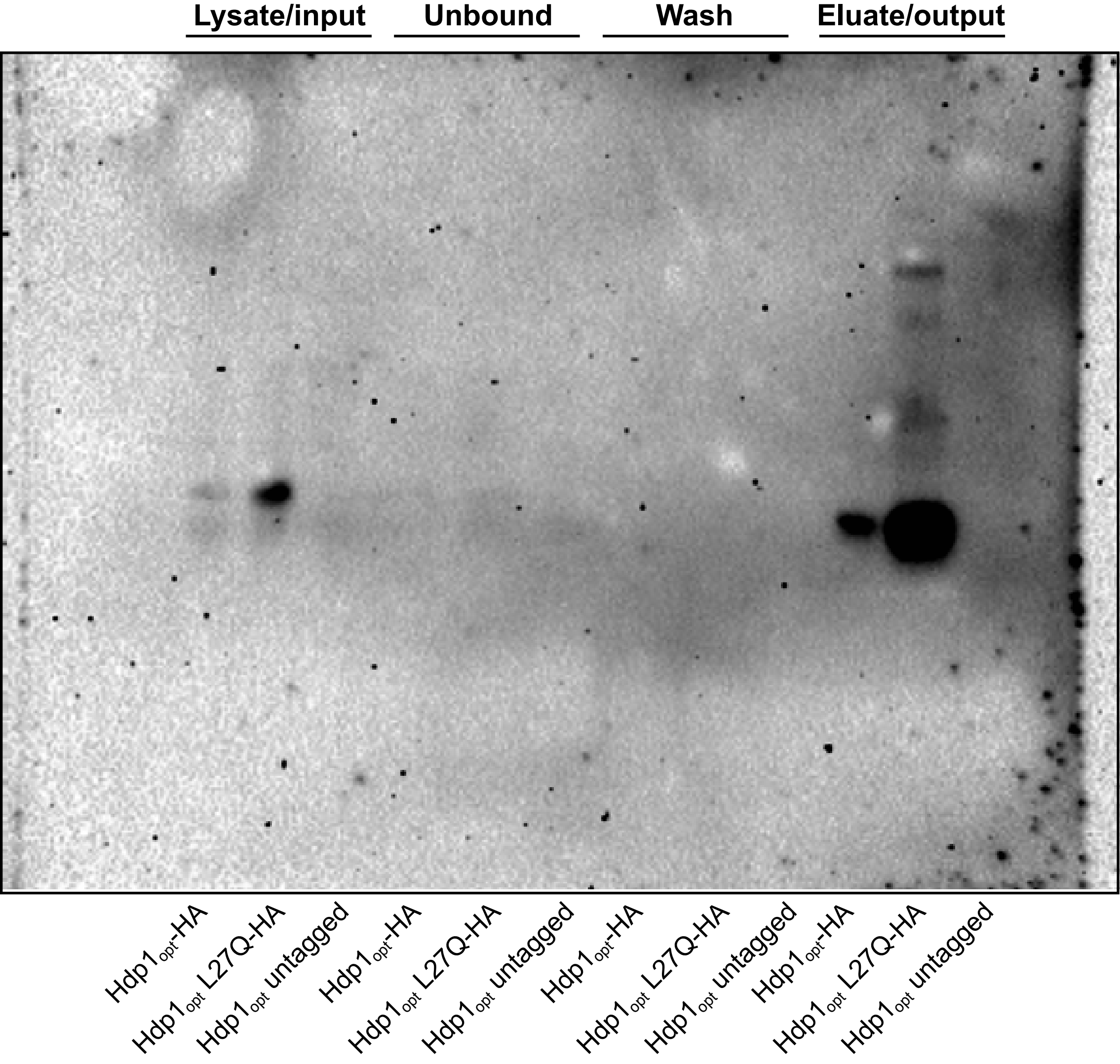
Lysate/input
Unbound
Wash
Eluate/output
Hdp1opt-HA
Hdp1opt L27Q-HA
Hdp1opt untagged
Hdp1opt-HA
Hdp1opt L27Q-HA
Hdp1opt untagged
Hdp1opt-HA
Hdp1opt L27Q-HA
Hdp1opt untagged
Hdp1opt-HA
Hdp1opt L27Q-HA
Hdp1opt untagged
